# Serum serotonin levels are elevated in patients with increased risk of rheumatoid arthritis

**DOI:** 10.3389/fmed.2022.1081814

**Published:** 2023-01-04

**Authors:** Lina Wirestam, Klara Martinsson, Alf Kastbom

**Affiliations:** Department of Biomedical and Clinical Sciences, Linköping University, Linköping, Sweden

**Keywords:** serotonin, rheumatoid arthritis, at-risk patients, early RA, biomarkers

## Abstract

**Background:**

Even though serotonin (5-HT) has been ascribed immunomodulatory features, very little is known about its role in chronic inflammatory diseases. Serotonin is implicated in inflammation and increased levels have been associated with progression of bone erosions in RA.

**Objective:**

To investigate serum serotonin levels in patients with increased risk of rheumatoid arthritis (RA) and patients with recent-onset disease. Moreover, we aimed to determine the prognostic value of serotonin for arthritis development and the disease course.

**Methods:**

Two prospective observational patient cohorts were studied; anti-citrullinated protein antibody (ACPA) -positive patients with musculoskeletal pain without clinical arthritis (*n* = 82) and patients with early RA (*n* = 412). Serotonin levels were measured by enzyme-linked immunosorbent assay (ELISA) in baseline serum samples from both cohorts, and longitudinally in at-risk individuals.

**Results:**

Compared to healthy controls (median 65 ng/ml), serotonin levels were significantly higher in both at-risk individuals (median 111 ng/ml, *p* < 0.0001) and patients with early RA (median 135 ng/ml, *p* < 0.0001). No significant differences were found between at-risk individuals and patients with early RA. At-risk individuals progressing to arthritis had similar levels as those not progressing, and no significant differences were seen over time. Baseline levels in early RA did not associate with mean 28-joint disease activity scores during 3 years follow-up.

**Conclusion:**

Serum serotonin levels are elevated both at, and prior to, onset of RA. However, increased serotonin is not prognostic for arthritis development or disease course.

## 1. Introduction

Increasing evidence suggest that early stages of rheumatoid arthritis (RA) occur outside the joint, and circulating autoantibodies, in particular anti-citrullinated protein antibodies (ACPAs), can be detected up to 10 years before diagnosis (1). Among individuals with ACPA who also experience arthralgia, 30–50% will develop arthritis within a few years (2, 3). In this prephase of RA, prognostic markers for arthritis development are of significant clinical value in order to achieve early and individualized treatment to prevent disease progression.

Serotonin has during the last decades been recognized as a peripheral hormone with immunomodulatory properties. The majority of the peripheral (i.e., outside the nervous system) serotonin is produced by intestinal enterochromaffin cells, and intriguingly, mucosal sites have emerged as a triggering site for RA development (4). Following release from enterochromaffin cells, serotonin enters the blood stream where it is taken up by platelets and stored in their dense granules until activation (5).

There is increasing interest in serotonin as an activator of inflammation and trigger of autoimmunity. For instance, elevated serotonin levels have been reported in RA patients compared to controls (6–8), and to associate with progression of bone erosions (9). Since serotonin appear increased in RA patients, we aimed to investigate if serotonin is elevated also prior to diagnosis. In addition, whether serotonin levels are prognostic for arthritis development in patients at increased risk of RA, or for the disease course among patients with recent-onset RA.

## 2. Materials and methods

### 2.1. Study subjects

Two prospective Swedish observational cohorts formed the basis of this study. Clinical patient characteristics are outlined in Table 1. The TIRx (extra-early rheumatology follow-up) cohort represents a population with increased risk of developing RA. Inclusion criteria were positive ACPA test (anti-CCP2) in clinical routine care and musculoskeletal pain of any kind and duration (10). Exclusion criteria were previous inflammatory rheumatic disease or corticosteroid treatment (oral or intra-articular) within 6 weeks prior to screening. A total of 82 patients without arthritis upon clinical examination at baseline were included, thus representing an at-risk population. During a median of 6 years follow-up, 39 (48%) of the patients developed arthritis, defined by clinical examination by an experienced rheumatologist (10).

**TABLE 1:** Baseline characteristics of study participants.

	At-risk individuals (TIRx) *n* = 82	Recent-onset RA (TIRA-2) *n* = 412	Controls
Age [median (range), years]	53.5 (18–76)	60.0 (18–87)	55 (18–72)
Sex [female (%)]	66 (81)	273 (66)	50 (50)
Rheumatoid factor positive [*n* (%)]	24 (29)	246 (60)	N/A
IgG ACPA positive [*n* (%)]	82 (100)	280 (69)	N/A

We also studied 412 early RA patients from the TIRA-2 (Timely Interventions in RA) cohort (11). Inclusion criteria were symptom duration ≥6 weeks but <12 months since the first joint swelling as judged by the patient. In addition, patients should fulfill four of seven of the 1987 revised American College of Rheumatology criteria for RA (12) (*n* = 387 [94%]) or experience morning stiffness for ≥60 min and symmetrical arthritis and small joint arthritis (fingers, wrists, or toes) (*n* = 25 [6%]). Radiographic damage was graded according to the Larsen method (13).

No participating individual had received disease-modifying anti-rheumatic drugs (DMARDs) prior to the initial blood sampling (baseline visit).

As controls, we recruited 100 blood donors who were age-matched for TIRx [mean age 55 (range 18–72) years, 50% women] from the Department of Transfusion Medicine at Linköping University Hospital.

Ethical permission has been granted; EPN-Linköping Dnr M168-05 (TIRA2), M220-09 (TIRx), and 2015/236-32 (controls).

### 2.2. Laboratory analyses

Serum samples, stored at −70°C until analysis, were analyzed in duplicates for serotonin by enzyme-linked immunosorbent assay (ELISA) according to the manufacturer’s instructions (ImmuSmol, Bordeaux, France).

Erythrocyte sedimentation rate (ESR), C-reactive protein (CRP) and platelet count were analyzed according to clinical routine practice at the respective participating rheumatology unit.

### 2.3. Statistical analysis

Mann–Whitney *U* test and Kruskal Wallis were used to test differences in levels between groups and Pearson’s chi-square was used for dichotomous variables. For correlation analyses, Spearman’s correlation was applied. Cox regression was used to test serotonin levels versus (vs.) progression to arthritis. Statistical analyses were performed with SPSS statistics version 26 (IBM, Armonk, NY, USA) or GraphPad Prism version 9 (GraphPad Software, La Jolla, CA, USA). Two-sided *p*-values < 0.05 were considered significant.

## 3. Results

### 3.1. Serotonin levels in at-risk individuals and early RA patients

**FIGURE 1:**
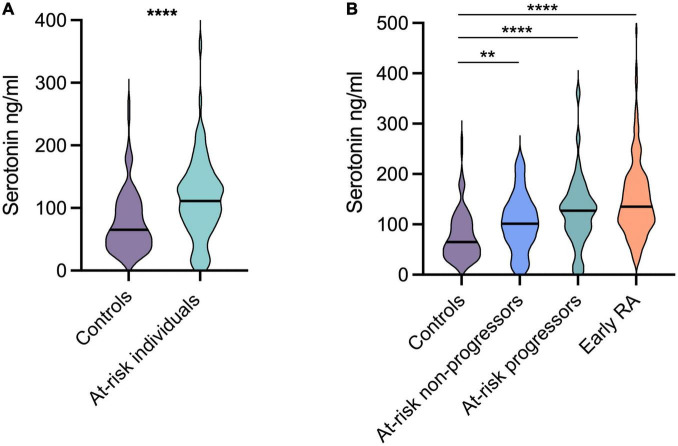
Serotonin levels in at-risk individuals and patients with early rheumatoid arthritis (RA). **(A)** Compared to healthy controls, the serotonin levels were significantly higher in at-risk individuals (median 65 ng vs. 111 ng/ml). **(B)** Patients with early RA had significantly higher levels (median 135 ng/ml) compared to healthy controls (median 65 ng/ml). The levels were comparable between at-risk individuals progressing and not progressing to arthritis (median 127 ng/ml and 102 ng/ml respectively). ****p < 0.0001, **p = 0.002.

Baseline serum serotonin levels were significantly higher in at-risk individuals (median 111 ng/ml) compared to healthy controls (median 65 ng/ml) *p* < 0.0001 (Figure 1A). The levels were comparable between patients progressing (median 127 ng/ml) vs. not progressing (median 102 ng/ml) to arthritis (Figure 1B). Patients with early RA also had significantly higher levels (median 135 ng/ml) compared to healthy controls (median 65 ng/ml, *p* < 0.0001; Figure 1B). No significant differences were found between at-risk individuals and patients with early RA. Neither were there any differences when comparing patients with early RA vs. at risk-individuals progressing to arthritis, or vs. at-risk individuals not progressing to arthritis (Figure 1B).

**FIGURE 2:**
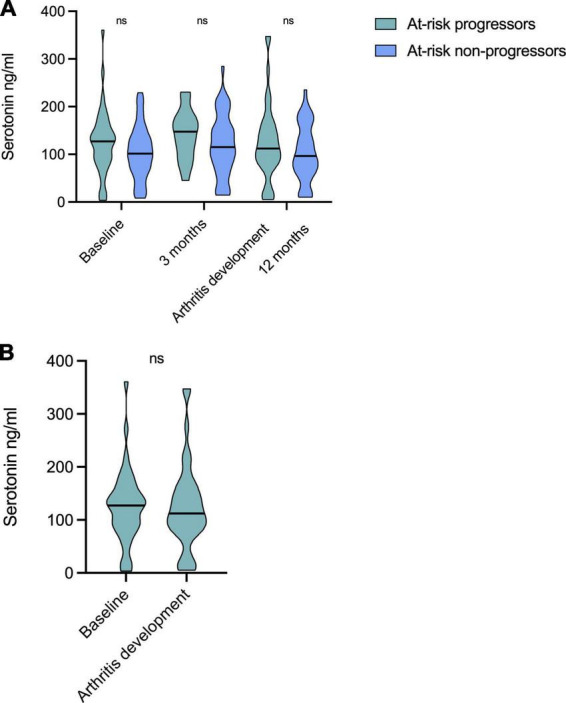
Longitudinal serotonin levels in at-risk individuals. **(A)** Serotonin levels were relatively stable over time and no differences were seen between any time points for patients progressing to arthritis, nor for non-progressors. Patients progressing to arthritis had numerically higher levels compared to those not progressing at all time points, but not significantly different. **(B)** Serotonin levels at baseline (median 127 ng/ml) did not differ to those at the time for arthritis development (median 112 ng/ml) among progressors. ns, non significant.

Longitudinal analyses in at-risk individuals revealed relatively stable serotonin levels over time (Figure 2A). At-risk patients progressing to arthritis had numerically higher median levels compared to those not progressing at all time points, but without statistical significance (baseline: median 127 vs. 102 ng/ml; 3 months; 147 vs. 115 ng/ml, time-point for arthritis development vs. 12 months; 112 vs. 97 ng/ml). Among progressors, serotonin levels at baseline (median 127 ng/ml) were similar to those at the time for arthritis development (median 112 ng/ml) (Figure 2B). Serotonin levels were not prognostic for arthritis development, as analyzed by Cox regression (*p* = 0.062).

### 3.2. Serotonin and laboratory signs of inflammation

In at-risk individuals, no statistically significant difference in baseline serotonin levels were found between patients with normal (*n* = 76, median 108 ng/ml) vs. raised CRP (*n* = 6, median 136 ng/ml) *p* = 0.36. Patients with raised ESR had lower serotonin (*n* = 21, median 73 ng/ml) compared to patients with normal ESR (*n* = 60, median 122 ng/ml), but not significant *p* = 0.055. Our serotonin analysis detects total serum content, including a potential proportion from platelets. Therefore, we investigated the association between serum serotonin and platelet count (rho = 0.15, *p* = 0.18). A total of 11 patients have elevated platelet count (i.e., above the reference range), but no significant difference regarding serotonin was seen when comparing patients with high (median 142 ng/ml) and normal (median 109 ng/ml) platelet count, *p* = 0.08.

No correlations were found between serotonin levels and CRP over time among at-risk individuals; baseline CRP (rho = 0.10, *p* = 0.38), after 3 months (rho = −0.15, *p* = 0.20) or after 12 months (rho = 0.14, *p* = 0.38). Analyzing ESR and serotonin over time revealed a significant correlation after 3 months (rho = −0.23, *p* = 0.04). Otherwise, no correlations were found; baseline ESR (rho = −0.13, *p* = 0.25) and after 12 months (rho = −0.12, *p* = 0.48).

### 3.3. Serotonin and disease course

In early RA, no correlations were found between baseline serotonin and disease activity (DAS 28) over time; Baseline DAS 28 (rho = −0.016, *p* = 0.75), after 3 months (rho = 0.008, *p* = 0.89) or after 36 months (rho = −0.002, *p* = 0.97). Serotonin did not correlate with baseline ESR (rho = 0.009, *p* = 0.86) or CRP (rho = −0.010, *p* = 0.84). There was no correlation between serotonin and baseline erosion defined by the Larsen score (rho = −0.018, *p* = 0.72) or after 36 months (rho = 0.077, *p* = 0.23). No differences in serotonin were seen in early RA patients positive vs. negative for ACPA (median 140 vs. 128 ng/ml, *p* = 0.33).

### 3.4. Serotonin levels vs. non-steroidal anti-inflammatory drugs and pain

At risk-individuals prescribed non-steroidal anti-inflammatory drugs (NSAIDs) at their baseline visit (*n* = 31) had slightly higher baseline serotonin levels (median 127 ng/ml) compared to those who were not prescribed NSAIDs (*n* = 51, median 97 ng/ml), but this was not statistically significant (*p* = 0.059). However, serotonin levels at 3 months among individuals with NSAID prescribed at their baseline visit were higher compared to those without prescription (152 ng/ml vs. 105 ng/ml, *p* = 0.012).

In the early RA cohort (143 prescribed vs. 264 not prescribed at baseline), this difference was borderline significant (median 145 ng/ml vs. 130 ng/ml, *p* = 0.05). When combining both cohorts, patients who were not prescribed NSAIDs still had significantly higher baseline serotonin levels compared to controls (median 127 ng/ml vs. 65 ng/ml, *p* < 0.0001).

No correlations were found between baseline serotonin levels and visual analog pain scales (VAS Pain) at baseline (rho = 0.039, *p* = 0.44), after 3 months (rho = −0.037, *p* = 0.49) or after 36 months (rho = 0.071, *p* = 0.20) in early RA. In at-risk individuals, we only had access to patient global assessment VAS, which did not associate with baseline serotonin levels (rho = 0.073, *p* = 0.52).

## 4. Discussion

In this study on carefully characterized patients in different phases of RA, we show for the first time that serum serotonin levels are increased already prior to the onset of RA. High serotonin levels have previously been reported in established RA (6–8), and now we extend this knowledge by showing that serotonin is elevated also in recent-onset RA. Interestingly, patients with early RA and at-risk individuals showed similar concentrations, indicating that serotonin may be involved in early triggering events rather than effector mechanisms. Platelets from patients with RA are shown to be more active compared to controls, and may be activated by ACPAs (14). However, no differences in serotonin levels were seen in early RA patients positive vs. negative for ACPA, and hence ACPA-mediated activation of platelets do not seem to be the cause of increased serum serotonin in the present study. Moreover, an inverse relationship between intra-cellular platelet serotonin levels and clinical disease activity has been observed in RA (15), and elevated platelet count during inflammation is a well-known phenomenon (16). We found no correlation between serotonin and platelet count, and patients with a platelet count above the reference range did not display elevated serotonin. Consequently, the raised serotonin is unlikely to be explained by an increased platelet number.

Centrally produced serotonin have been studied in many pain-related disorders, but the peripheral actions from serotonin differ from the central actions. Serotonin released from platelets and mast cells following injury and inflammation in the periphery can intensify pain by sensitization of nerve fibers (17, 18), and high serum concentrations have been associated with temporomandibular joint pain in RA (7). The elevated levels in early RA patients showed no association with the degree of pain.

Serotonin has been linked to bone loss. Bernardes et al. (19) could not, in contrast to us, find any differences in serum serotonin levels between patients and controls, but instead reported an inverse weak association with femur bone density in postmenopausal RA women. An inverse relation between serum serotonin and erosion in the temporomandibular joints has also been reported (9), as well as the use of serum serotonin as a prognostic marker of radiologic outcome in RA (20). We could not confirm any association between serotonin and radiographic joint damage, in early RA.

Genetic polymorphisms within the serotonin receptor HTR2A gene have been shown to associate with RA susceptibility (21, 22). Moreover, the density of serotonin 5-HT2A receptors is decreased in RA patients and correlated with a more severe disease (23), suggesting possible links between the serotonergic system and development of the disease. Possibly, decreased receptor expression could lead to an increased proportion of free serotonin, and should be addressed in upcoming studies.

There is a possibility that pharmacotherapy influences serotonin levels. At-risk individuals prescribed NSAIDs at their first visit had higher serotonin levels compared to those without such prescription, but this was not statistically significant. Unfortunately, we lack data on the patients’ use of NSAID without prescription before their first visit. However, the serotonin levels after 3 months were higher among patients with baseline prescription, possibly affecting future levels. NSAID have no impact on platelet count but impairs platelet degranulation and activation (24). Serotonin is synthesized from tryptophan, which may also be metabolized by the enzyme indoleamine 2,3-dioxygenase (IDO) into kynurenine. NSAID may decrease IDO, thereby theoretically increase the availability of tryptophan for serotonin production (25). Although NSAID supposedly could increase serotonin levels, patients without NSAID prescribed still had considerable higher levels compared to healthy controls. Nevertheless, the absence of pre-screening NSAID data is a limitation of our study, in addition to the lack of controls with other inflammatory or non-inflammatory conditions. Further, the at-risk cohort size did not allow subgrouping of patients.

In conclusion, serotonin serum levels are increased both at and prior to onset of RA but is not prognostic for arthritis development or disease activity over time. Although we find no clinical value of serum serotonin analysis in patients at risk or with early RA, the elevated levels call for future studies to elucidate the role of the serotonin system in RA development.

## Data availability statement

The raw data supporting the conclusions of this article will be made available by the authors, without undue reservation.

## Ethics statement

The studies involving human participants were reviewed and approved by the EPN Linköping. The patients/participants provided their written informed consent to participate in this study.

## Author contributions

LW: designing of the project, acquisition and analyses of patient data, interpretation of results, and writing of the manuscript. KM and AK: interpretation of results and writing of the manuscript. All authors contributed to the article and approved the submitted version.
